# Biodistribution and Immunogenicity of Allogeneic Mesenchymal Stem Cells in a Rat Model of Intraarticular Chondrocyte Xenotransplantation

**DOI:** 10.3389/fimmu.2017.01465

**Published:** 2017-11-06

**Authors:** Maribel Marquina, Javier A. Collado, Magdiel Pérez-Cruz, Pablo Fernández-Pernas, Juan Fafián-Labora, Francisco J. Blanco, Rafael Máñez, María C. Arufe, Cristina Costa

**Affiliations:** ^1^Infectious Diseases and Transplantation Division, Institut d’Investigació Biomèdica de Bellvitge – IDIBELL, Bellvitge University Hospital, ICS, L’Hospitalet de Llobregat, Barcelona, Spain; ^2^Cellular Therapy and Medicine Regenerative Group, Department of Medicine, Instituto de Investigación Biomédica de A Coruña (INIBIC), Complexo Hospitalario Universitario de A Coruña (CHUAC), Sergas, Universidade da Coruña, As Xubias, A Coruña, Spain; ^3^Grupo de Proteómica-ProteoRed/Plataforma PBR2-ISCIII, Servicio de Reumatología, Instituto de Investigación Biomédica de A Coruña (INIBIC), Complexo Hospitalario Universitario de A Coruña (CHUAC), Sergas, Universidade da Coruña, As Xubias, A Coruña, Spain

**Keywords:** xenotransplantation, chondrocytes, alloimmunity, mesenchymal stem cells, immune rejection

## Abstract

Xenogeneic chondrocytes and allogeneic mesenchymal stem cells (MSC) are considered a potential source of cells for articular cartilage repair. We here assessed the immune response triggered by xenogeneic chondrocytes when injected intraarticularly, as well as the immunoregulatory effect of allogeneic bone marrow-derived MSC after systemic administration. To this end, a discordant xenotransplantation model was established by injecting three million porcine articular chondrocytes (PAC) into the femorotibial joint of Lewis rats and monitoring the immune response. First, the fate of MSC injected using various routes was monitored in an *in vivo* imaging system. The biodistribution revealed a dependency on the injection route with MSC injected intravenously (i.v.) succumbing early after 24 h and MSC injected intraperitoneally (i.p.) lasting locally for at least 5 days. Importantly, no migration of MSC to the joint was detected in rats previously injected with PAC. MSC were then administered either i.v. 1 week before PAC injection or i.p. 3 weeks after to assess their immunomodulatory function on humoral and adaptive immune parameters. Anti-PAC IgM and IgG responses were detected in all PAC-injected rats with a peak at week 2 postinjection and reactivity remaining above baseline levels by week 18. IgG2a and IgG2b were the predominant and long-lasting IgG subtypes. By contrast, no anti-MSC antibody response was detected in the cohort injected with MSC only, but infusion of MSC before PAC injection temporarily augmented the anti-PAC antibody response. Consistent with a cellular immune response to PAC in PAC-injected rats, cytokine/chemokine profiling in serum by antibody array revealed a distinct pattern relative to controls characterized by elevation of multiple markers at week 2, as well as increases in proliferation in draining lymph nodes. Notably, systemic administration of allogeneic MSC under the described conditions did not diminish the immune response. IL-2 measurements in cocultures of rat peripheral blood lymphocytes with PAC indicated that PAC injection induced some T-cell hyporesponsiveness that was not enhanced in the cohorts additionally receiving MSC. Thus, PAC injected intraarticularly in Lewis rats induced a cellular and humoral immune response that was not counteracted by the systemic administration of allogeneic MSC under the described conditions.

## Introduction

There is a major clinical need to find curative therapies for the repair of articular cartilage defects secondary to trauma or diseases, such as osteoarthritis (OA). The use of xenogeneic cells could greatly benefit the development of this treatment modality as they could be obtained in sufficient quantity following protocols with high quality and control (1). Notably, these cells could be genetically engineered to match the needs of every particular application in clinical practice (2, 3). Xenogeneic porcine chondrocytes are differentiated cells with the capacity to form hyaline cartilage in *in vitro* and *in vivo* models (4, 5). However, they could be potentially rejected by cellular and humoral mechanisms, which have not been fully characterized. The most relevant preclinical models for xenotransplantation of porcine cartilage have shown rejection in non-articular sites (6, 7). Subsequent studies led to the identification of key molecules and pathways responsible for triggering an immune response against porcine chondrocytes (8–10). Although their participation in articular cartilage rejection remains unclear, genetic engineering approaches could be applied to selected targets once this process is better understood (11). The high impact of articular cartilage injury and disease raises the need to consider the characteristics of the joint in terms of structure and immunobiology for the design of preclinical studies.

Mesenchymal stem cells (MSC) are the focus of research for cartilage repair as MSC meet many requirements for their use in cell therapies (12–14). MSC display immune modulation and promotion-of-tissue-regeneration properties. Most studies and clinical trials have focused on using MSC as an alternative to chondrocytes for direct implantation in the cartilage defect in combination with scaffolds or gels (12–14). However, evidence of superiority of these approaches over autologous chondrocyte implantation (ACI) or related techniques is currently lacking. ACI requires the harvest of cartilage from the patient for subsequent isolation, expansion, and transplantation of chondrocytes into the defect, implying limitations in cell number and quality. Thus, the combination of MSC and chondrocytes *in situ* is also being considered (12). Interestingly, MSC are being assessed for the treatment of OA in clinical trials [mostly based on intraarticular (i.a.) injection into the affected joint] with promising results (14, 15). The benefits of these MSC-based therapies for OA are basically attributed to their anti-inflammatory and immunosuppressive actions.

Mesenchymal stem cells, with some variations depending on the species and tissue of origin, exert immunomodulatory activities through multiple pathways that predominantly result in inhibition of both innate and adaptive immune responses (16–19). In particular, MSC suppress proliferation and cytokine release of both CD4^+^ and CD8^+^ T lymphocytes in an MHC-independent manner, as well as promote a shift from Th1/Th17 toward Th2 phenotype and the generation of regulatory T cells (16). An inhibitory effect on B cells, NK cells, monocytes/macrophages, dendritic cells, and neutrophils has also been reported (16). Thus, allogeneic MSC have demonstrated therapeutic efficacy and pose advantages versus autologous cells in terms of processing, quantity, and control (16). The administration route is also relevant for MSC to exert the immunosuppressive activity *in vivo* (19). Systemic administration is favored in cases of autoimmunity, graft-versus-host disease and frequently for facilitating tolerance of vascularized allografts (16, 18–20). Nevertheless, MSC administration does not always produce the desired tolerogenic effect as they can be also immunogenic depending on the setting (17, 18, 20–22).

In the present experimental study, we assessed whether xenogeneic porcine chondrocytes transplanted into the rat joint trigger an immune response. This xenotransplantation model was designed to provide a highly stringent setting for preclinical testing of future xenogeneic therapies for articular cartilage. In this work, we used this model to study the immunoregulatory effects of allogeneic MSC administered systemically as a single dose either 1 week before or 3 weeks after the chondrocytes.

## Materials and Methods

### Porcine Articular Chondrocytes (PAC) Isolation and Culture

Porcine articular chondrocytes were isolated previously from non-transgenic pigs according to previously published methods (7, 10), frozen at passages 0 and 1 in DMEM mixed with 10% DMSO and 50% FBS (both v/v) and stored in liquid nitrogen until use. PAC were thawed and expanded in DMEM with 10% FBS (v/v), 100 IU/ml penicillin–100 mg/ml streptomycin, and 25 µg/ml endothelial cell growth supplement (Millipore). All media and antibiotics were from Life Technologies and FBS from Biological Industries (Kibbutz Beit Haemek, Israel).

### Isolation, Culture, Genetic Engineering, and Characterization of Rat MSC

Mesenchymal stem cells were isolated from bone marrow of 14-day-old male Wistar rats (Animal Service, CHUAC) according to previously published procedures (23). The study protocols were approved by the Animal Ethical Committee of Galicia. Briefly, the ends of femurs were cut away, and bone marrow was extruded by flushing with 5 ml D-Hank’s solution supplemented with antibiotics. Marrow suspensions were dispersed by pipetting, filtered and centrifuged as previously described (23). Cell pellets from four rats were pooled in RPMI with 10% FBS and antibiotics, seeded and cultured in 100-cm^2^ dishes (TM Nunclon). Adherent cells grown to 70% confluence were defined as passage-0 MSC and expanded for two passages for characterization before use (23).

Mesenchymal stem cells expressing firefly luciferase (LUC-MSC) were generated by lentiviral infection using Lenti-X™ Lentiviral expression System (Clontech). Viral supernatants were obtained from 293 T cells transfected with pGL 4.14-Luc2 (Promega) following standard procedures and stored at 4°C until transduction. MSC at 70% confluency were incubated sequentially with viral supernatants, washed and cultured for 2 days before selection with 1 µg/ml puromycin (Clontech) for 5 days. After selection, LUC-MSC were allowed to recover in complete media. Their function was tested *in vitro* by incubating the LUC-MSC and negative controls in phosphate-buffered saline (PBS) with 150 µg/ml of firefly D-luciferin (Biosynth AG, Staad, Switzerland) followed by bioluminescence imaging in an IVIS Lumina XR imaging system using Living Image 4.3.1 software (Caliper Life Sciences, Waltham, MA, USA).

Inducible nitric oxide synthase (iNOS) was detected by flow cytometry in MSC and murine fibroblasts (3T3L1) cultured normally or after exposure to 10 ng/ml lipopolysaccharides (Sigma-Aldrich) for 24 h. During harvest, cells were washed with PBS (MP Biomedicals), then fixed in 4% paraformaldehyde (w/v) (Sigma-Aldrich) for 10 min, washed twice, and pre-blocked with 2% (v/v) rat serum (Life Technologies). Cells were incubated with rabbit polyclonal antibody anti-iNOS (Abcam, Cambridge, UK) for 1 h, washed and incubated with goat anti-rabbit immunoglobulin-phycoerythrin (PE) (Dako, Glostrup, Denmark) for 1 h for measurements in a FACSAria using DIVA software (BD Biosciences).

### Animal Studies

Four different animal experiments were carried out that involved i.a. injection of PAC into the right femorotibial joint of Lewis rats (summarized in Figure S1 in Supplementary Material). The protocols were approved by the Ethics Committee of the University of Barcelona and the government of Catalonia. Experiments were performed following a group sequential design starting with a pilot with a low number of rats per group before proceeding with a larger study if considered appropriate (24). Male rats (weighing 200–250 g) were purchased from Harlan (Barcelona, Spain) and kept under controlled conditions of light and temperature with food and water *ad libitum* at the animal facilities of the University of Barcelona. Body weight and animal well-being were monitored throughout the study. In preparation for cell injection, PAC and MSC were washed thoroughly by centrifugation to remove the FBS, resuspended in DMEM with antibiotics (vehicle), and kept at room temperature. Cells were mixed with 15 µl of vehicle/joint for i.a. injections, 200 μl/rat for intraperitoneal (i.p.) injections, and 100 μl/rat for intravenous (i.v.) injections. All injections were performed in animals anesthetized with isofluorane.

For the MSC localization studies (Figure S1A in Supplementary Material), we used a total of 18 rats distributed in six experimental cohorts (*n* = 3/group). Control rats received a single injection with 2.5 × 10^6^ cells/kg body weight of LUC-MSC i.a., i.v., or i.p. The other rats were subjected to one of three different regimes involving pretreatment: (1) one cohort received DMEM i.a. followed by i.a. injection of LUC-MSC (2.5 × 10^6^ cells/kg body weight) 2.5-weeks later, (2) and (3) the others were injected i.a. with 3 × 10^6^ PAC and 2.5 weeks later with the described amount of LUC-MSC, either i.v. or i.p. The follow-up by bioluminescence imaging was established at 2 and 24 h, and at 4 and 5 days after MSC injection in an IVIS Lumina XR imaging system using Living Image 4.3.1 software. For each determination, the rat was injected i.p. with 200 mg/kg of firefly D-luciferin and 15 min later was analyzed in the IVIS chamber under anesthesia with isofluorane.

In a pilot experiment assessing the immune response (Figure S1B in Supplementary Material), six male rats were equally distributed between two experimental groups to receive either PAC i.a. (cohort PAC, *n* = 3) or MSC i.v. and PAC i.a. (cohort MSC + PAC, *n* = 3). Injection of 3 × 10^6^ PAC was considered time 0, whereas MSC (2.5 × 10^6^ cells/kg body weight) were injected i.v. 1 week before the PAC. The serum antibody titers were monitored at 2, 5, 10, and 15 weeks. Cellular responses of isolated peripheral blood lymphocytes (PBL) were determined by coculture assay at 5 weeks post-PAC injection. One rat of the MSC + PAC group died after week 11. The loss was preceded by an arrest in growth that was detected at week 10, whereas the rest of rats continued to grow throughout the study.

In another experiment (Figure S1C in Supplementary Material), 22 rats were distributed between four experimental groups to receive either DMEM i.a. (cohort DMEM, *n* = 5), PAC i.a. (cohort PAC, *n* = 6), MSC i.p. (cohort MSC, *n* = 5) or PAC i.a. and MSC i.p. (cohort PAC + MSC, *n* = 6). Injection of 3 × 10^6^ PAC was considered time 0, whereas MSC (2.5 × 10^6^ cells/kg body weight) were injected 3 weeks later. The immune response was monitored at 2, 5, 10, and 18 weeks after PAC injection by measurement of serum anti-PAC antibody titers and cytokine profile at 2 and 5 weeks. Body weight was measured at the various time points. When indicated, one additional milliliter of blood was obtained from each of the five rats/cohort used for isolation of PBL and conducting coculture assays. For follow-up of the anti-MSC antibody response, the time points were adapted to consider the time of MSC injection as time 0, and the 5, 10, and 18 weeks post-PAC injection became, respectively, 2, 7, and 15 weeks after MSC injection.

An additional separate study was performed with 18 rats to assess the immune response at the level of draining lymph nodes (Figure S1D in Supplementary Material), Rats were equally distributed between experimental groups to receive either DMEM i.a. (*n* = 6) and 3 × 10^6^ PAC i.a. (*n* = 6) or MSC i.v. (2.5 × 10^6^ cells/kg body weight) 1 week before 3 × 10^6^ PAC i.a. (*n* = 6). Two weeks after the injection, the popliteal lymph nodes were harvested for lymphocyte isolation.

### Detection of Antibody Responses

The antibody response was assessed by flow cytometry using PAC or MSC as target cells and rat serum diluted as indicated for reactivity measurements. After incubation for 30 min at 4°C, anti-rat secondary antibodies [PE-conjugated goat F(ab′)2 anti-rat IgM and FITC-conjugated goat F(ab′)2 anti-rat IgG (Beckman Coulter Inc., Fullerton, CA, USA)] were added for further incubation at 4°C. In a separate set of experiments, PAC were incubated with the same sera (at 1%) up to week 10 to detect the different anti-PAC IgG subtypes using anti-rat secondary antibodies [mouse anti-rat IgG1, IgG2a, IgG2b, and IgG2c mAbs-FITC (Southern Biotech Inc., Birmingham, AL, USA)]. In both cases, cells were finally washed with PBS and analyzed by flow cytometry (Beckman Coulter Gallios™, Kaluza^®^ Analysis Software).

### Lymphocyte Isolation and Analysis

Peripheral blood lymphocytes were isolated from 1.5 ml of rat blood by Ficoll gradient followed by 2-h incubation at 37°C in RPMI/10% FBS to remove adherent cells. For isolation from lymph nodes, these were submitted to mechanical disruption into PBS/10% FBS and left to settle to remove large debris. Supernatants were transferred to clean tubes, washed by centrifugation and cell pellets resuspended in RPMI/10% FBS. In both cases, supernatants were recovered for cell counting in a hemocytometer in preparation for the assays.

T-cell activation was determined for individual rats in cocultures adapted from previously reported techniques (8, 25). Briefly, confluent PAC were incubated at 37°C in 96-well plates with PBL at 10:1 effector/target ratio. Controls of each cell type cultured alone were included. For additional activation, concanavalin A (ConA, Sigma) was added to selected wells at a final concentration of 10 µg/ml 30 min after initiating the coculture. Culture supernatants were harvested 24 h later, and IL-2 secretion was measured using Rat IL-2 ELISA kit (RayBiotech, Inc., Norcross, GA, USA).

For characterization, isolated lymphocytes were stained with anti-rat CD4-FITC and CD8-FITC antibodies alone or in combination with APC-conjugated anti-mouse Ki-67, PE-conjugated anti-Foxp3 or isotype control (all Miltenyi Biotec) for two-color analysis. The FoxP3 Staining Buffer Set (Miltenyi) was used for permeabilization and fixation. Cell surface staining of a gated population of lymphocytes was then measured in a Gallios flow cytometer.

### Cytokine/Chemokine Assays

Serum levels of 34 protein markers (Table 1) were assessed for most rats at baseline, week 2 and 5 postinjection by antibody array (Rat Cytokine Antibody Array G2, RayBiotech) with the technical support of tebu-bio (Le Perray-en-Yvelines, France). Final data corresponded to the average intensity of duplicate spots with the system’s background subtracted and normalized using internal controls.

**Table 1:** IL-2 secretion by PBL isolated from PAC and MSC + PAC rats cultured alone or with PAC for 24 h.

Time and number of rats	Cohorts	IL-2 (pg/ml) (mean ± SEM) and condition			
		PBL	PBL + ConA	PBL + PAC	PBL + PAC + ConA
5 weeks (*n* = 3)	PAC	89 ± 35	76 ± 31	53 ± 33	118 ± 53
	MSC + PAC	193 ± 68	128 ± 27	148 ± 47	274 ± 86

*PBL from the indicated number of rats were isolated and cultured separately in triplicates. Activation of the primary signal was conducted with ConA when indicated*.

*MSC, mesenchymal stem cells; PAC, porcine articular chondrocytes; PBL, peripheral blood lymphocytes*.

### Statistical Analysis

Data are presented as mean and SEM. Non-parametric statistical tests were used due to non-normal distribution of data. Statistical analysis was performed with the Statistical Package for Social Sciences (version 12.0, SPSS Inc., IL, USA) using Wilcoxon rank-sum test or the Mann–Whitney *U* test (called Mann–Whitney *U* thereafter) when comparing two groups of independent samples and Wilcoxon signed-rank test (called Wilcoxon thereafter) for the comparison between follow-up and baseline values. Differences were considered statistically significant at *p* ≤ 0.05.

## Results

### MSC Distribution following Different Administration Routes

**Figure 1:**
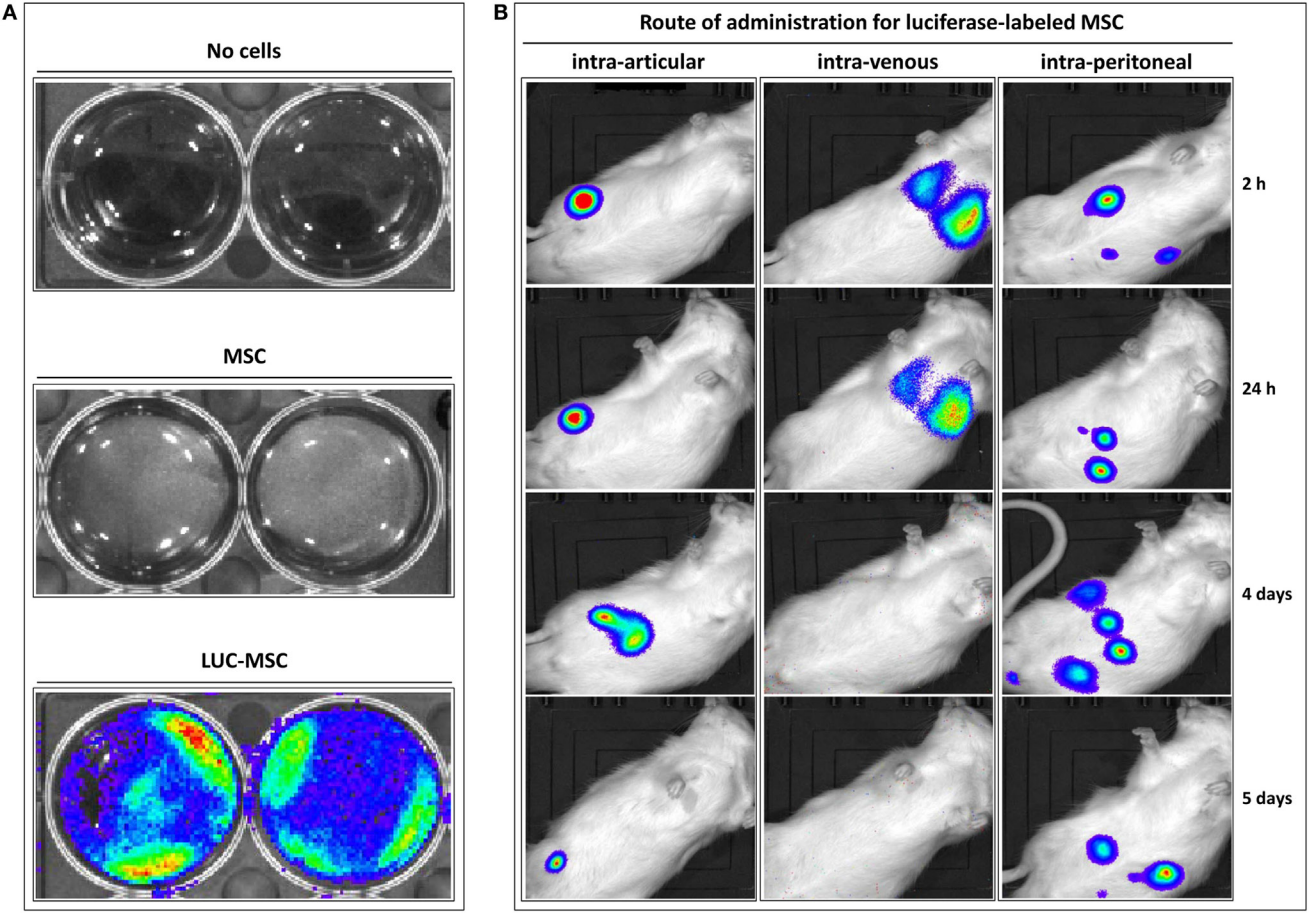
Distribution of allogeneic mesenchymal stem cells (MSC) in Lewis rats previously injected intraarticularly (i.a.) with porcine articular chondrocytes (PAC). Bioluminescence imaging was performed in an IVIS Lumina XR using Living Image 4.3.1 software. Pictures were taken with 1-min exposure to ensure detection. **(A)** Mesenchymal stem cells expressing firefly luciferase (LUC-MSC), genetically modified to express luciferase, and negative controls were incubated in phosphate-buffered saline with firefly D-luciferin for function confirmation. **(B)** Follow-up of biodistribution of LUC-MSC using different injection routes as indicated. Control rats injected with LUC-MSC i.a. did not receive PAC, the remaining animals were injected i.a. with PAC 2.5 weeks before MSC injection (Figure S1A in Supplementary Material).

The distribution of allogeneic MSC in Lewis rats previously injected i.a. with PAC was firstly studied (Figure S1A in Supplementary Material). To this purpose, bone marrow-derived MSC isolated from Wistar rats were genetically modified to express luciferase. These LUC-MSC, but not unmodified MSC, produced good bioluminescence signals when assayed *in vitro*, which confirmed activity in the presence of luciferin (Figure 1A). *In vivo*, the biodistribution was highly dependent on the injection route (Figure 1B). Control rats (without PAC i.a.) injected with LUC-MSC i.a. showed photon signals localized at the injection site for up to 5 days, although with reduced signal over time. No diffusion of the signal was observed for any of the rats in this group that would indicate leakage from the injection site for the first 24 h. In the other cohorts shown in Figure 1B, injection of PAC i.a. before MSC did not lead to MSC recruitment into the joint. The i.v. route led to MSC entrapment in the lungs with predominance of one lung over the other. The bioluminescence intensity was reduced by 24 h in all cases in this cohort and disappeared completely by day 4. On the contrary, MSC administered i.p. remained alive longer and were detected in the abdomen during the 5-day study period. Notably, no photon signals were observed at the joint area at any time point or animal following the i.v. or i.p. routes. Rats receiving MSC via these routes without prior PAC injection displayed an identical pattern (data not shown).

### Injection of PAC i.a. Induced a Xenogeneic Antibody Response Which Was Not Reduced by MSC Administered Systemically

**Figure 2:**
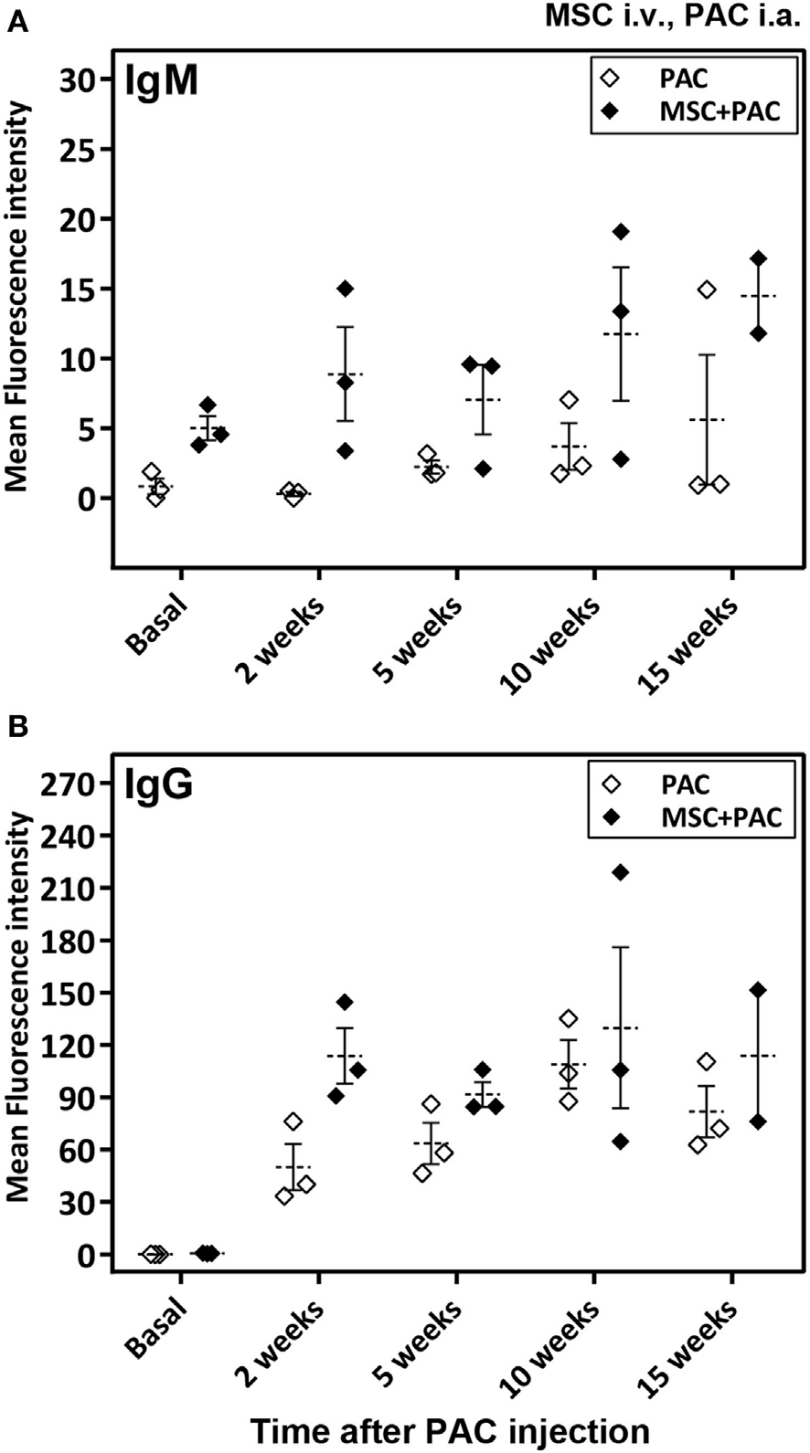
Antibody response in Lewis rats injected intraarticularly (i.a.) with porcine articular chondrocytes (PAC) only or pretreated with mesenchymal stem cells (MSC) intravenously (i.v.). A scheme of the experimental design is shown in Figure S1B in Supplementary Material. In particular, one cohort received only PAC i.a., whereas the other cohort was injected with MSC i.v. 1 week before PAC i.a. injection (MSC + PAC) and both followed-up for 15 weeks. Anti-PAC IgM **(A)** and IgG **(B)** antibody reactivity was determined by flow cytometry for all rat sera (0.6% final dilution) collected at baseline and at 2, 5, 10, and 15 weeks after PAC injection. The mean ± SEM of mean FL-1 fluorescence intensity after subtracting the background (reactivity of secondary antibody alone) is shown (*n* = 3 for PAC and MSC + PAC cohorts).

As a major goal in our study, we sought to assess whether i.a. injection of PAC induced an immune response. As a second objective, additional experimental groups were included to study the effect of MSC systemic administration. The MSC utilized were confirmed to express iNOS at higher proportion than fibroblasts in stimulated conditions (Figure S2A in Supplementary Material). In an initial pilot experiment, we compared the immune response induced in a small number of rats either injected i.a. with PAC only or pretreated with MSC i.v. 1 week before the PAC injection (MSC + PAC cohort) (Figures S1B and 2B in Supplementary Material), An elicited anti-PAC IgM and IgG response was observed in all rats after i.a. injection of PAC (Figure 2). However, pretreatment with MSC i.v. did not reduce the antibody titers. In fact, the IgG antibody response may have been enhanced in MSC + PAC rats (Figure 2B). Likewise, MSC pretreatment seamed to increase overall T-cell reactivity in a coculture assay conducted to assess the cellular response using PAC and PBL isolated from each rat (Table 1). Although the low sample size did not allow statistical analyses for confirmation of these preliminary results, we chose to stop the experiment to avoid using more animals with these potential undesired effects.

The main experiment was designed to study the immune response to PAC-injected i.a. compared with a cohort receiving vehicle alone (DMEM), as well as the effect of MSC on this response once initiated (Figure S1C in Supplementary Material). To this end, MSC were administered i.p. 3 weeks after PAC injection in a PAC + MSC cohort. Notably, no deleterious effects were observed for any of the treatments (PAC or MSC) on the well-being and body weight of the animals throughout this experiment (Figure S2C in Supplementary Material). To monitor the humoral response, anti-PAC antibody titers were determined in all cohorts up to 18 weeks. Anti-MSC antibody reactivity was also measured for the DMEM and MSC cohorts. The cellular response was studied by assessing the serum cytokine/chemokine profile and setting up coculture assays with PBL isolated from each rat at selected time points.

**Figure 3:**
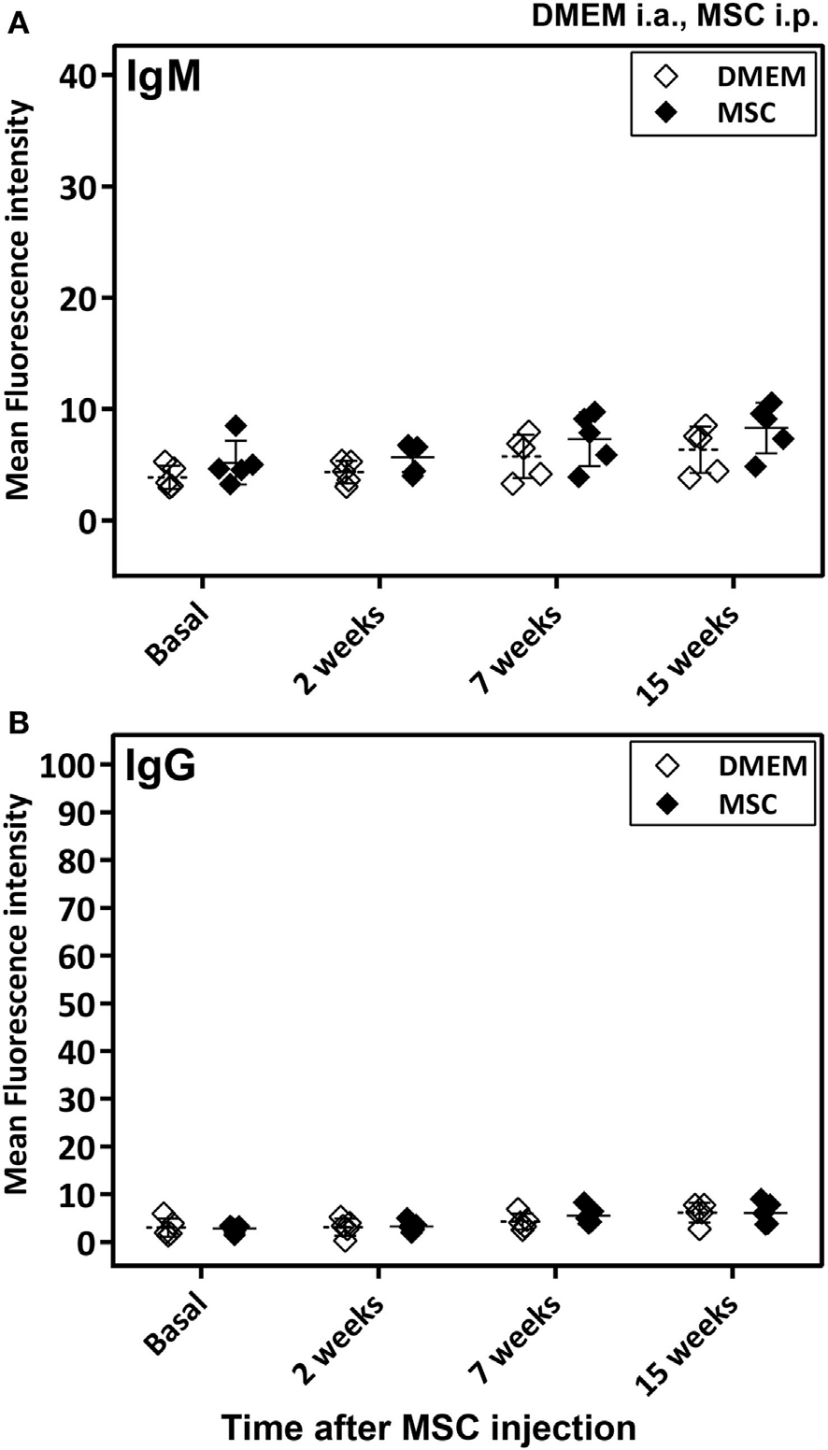
Antibody response in Lewis rats injected intraperitoneally (i.p.) with mesenchymal stem cells (MSC). The study included samples from the DMEM and MSC cohorts described in Figure S1C in Supplementary Material. In particular, the MSC cohort was injected i.p. with MSC and followed-up for 15 weeks at the indicated time points in this figure, whereas the control cohort was injected with DMEM i.a. 5 weeks before the 2-week time point. Anti-MSC IgM **(A)** and IgG **(B)** antibody reactivity was determined by flow cytometry for all rat sera (0.6% final dilution) collected at baseline and at 2, 7, and 15 weeks after MSC injection. The mean ± SEM of mean FL-1 fluorescence intensity after subtracting the background (reactivity of secondary antibody alone) is shown (*n* = 5). No significant differences were detected when results of the two cohorts were compared using the Mann–Whitney *U* test.

Notably, no anti-MSC antibody (IgM or IgG) reactivity was detected in sera of rats injected with MSC over controls for the duration of the study (up to 15 weeks after MSC injection) (Figure 3).

**Figure 4:**
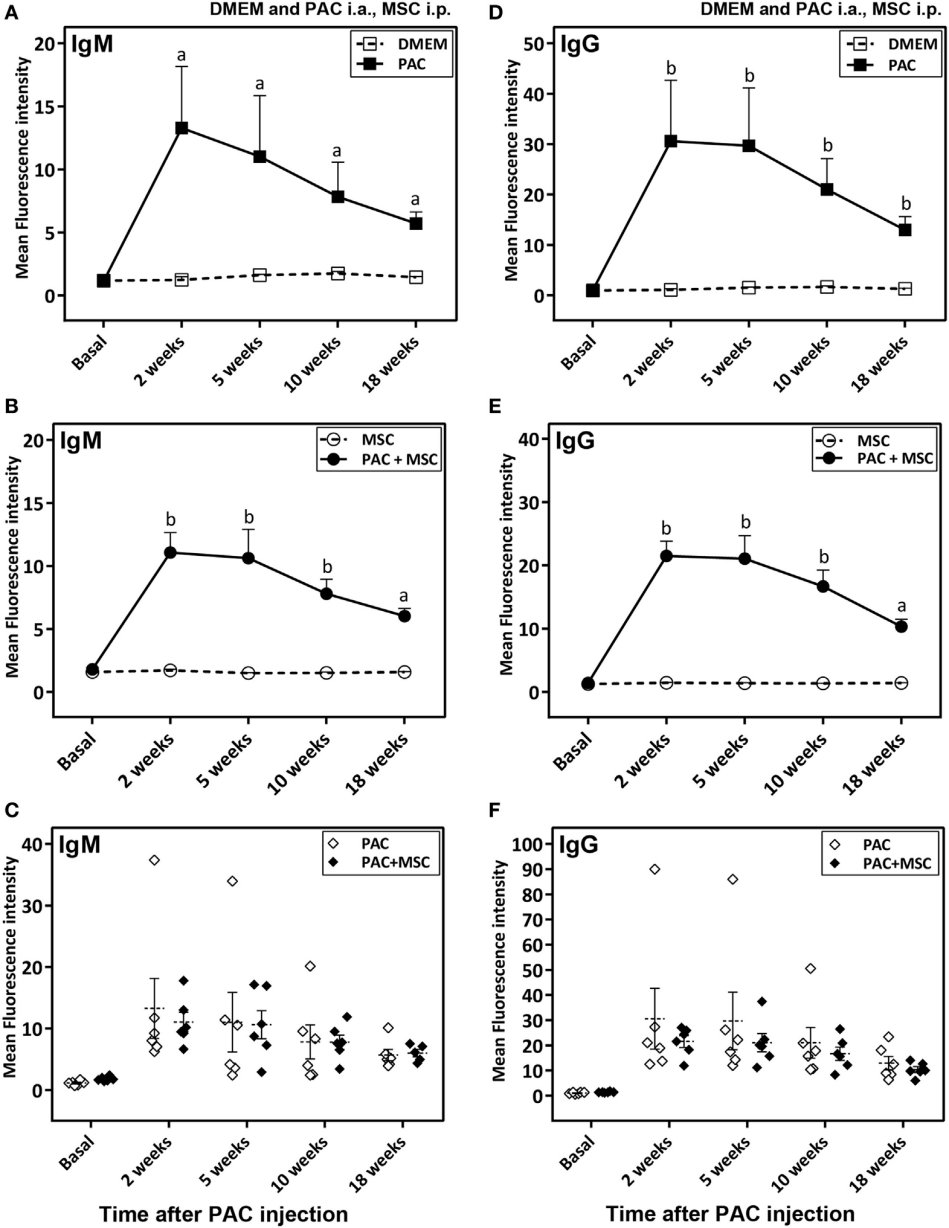
Antibody response in Lewis rats injected intraarticularly (i.a.) with porcine articular chondrocytes (PAC) only or posttreated with mesenchymal stem cells (MSC) intraperitoneally (i.p.). A scheme of the experimental design comprising four different groups is shown in Figure S1C in Supplementary Material. In particular, one cohort received only DMEM i.a., another only PAC i.a., another was injected with MSC i.p. 3 weeks after PAC i.a. injection, whereas the corresponding control received only MSC i.p. at the same time. All were followed-up for 18 weeks. Anti-PAC IgM **(A–C)** and IgG **(D–F)** antibody reactivity was determined by flow cytometry for all rat sera (0.5% final dilution) collected at baseline and at 2, 5, 10, and 18 weeks after PAC injection. The mean ± SEM of mean FL-1 fluorescence intensity after subtracting the background (reactivity of secondary antibody alone) is shown (*n* = 5 for DMEM and MSC cohorts; *n* = 6 for PAC and PAC + MSC cohorts). Statistically significant differences were observed using the Mann–Whitney *U* test for the 2-week time point, but not the baseline, for both IgM and IgG relative to corresponding levels of the control cohort (^a^*p* < 0.01, ^b^*p* < 0.005). No significant differences were detected when results of the PAC and PAC + MSC cohorts were compared using the Mann–Whitney *U* test **(C,F)**.

**Figure 5:**
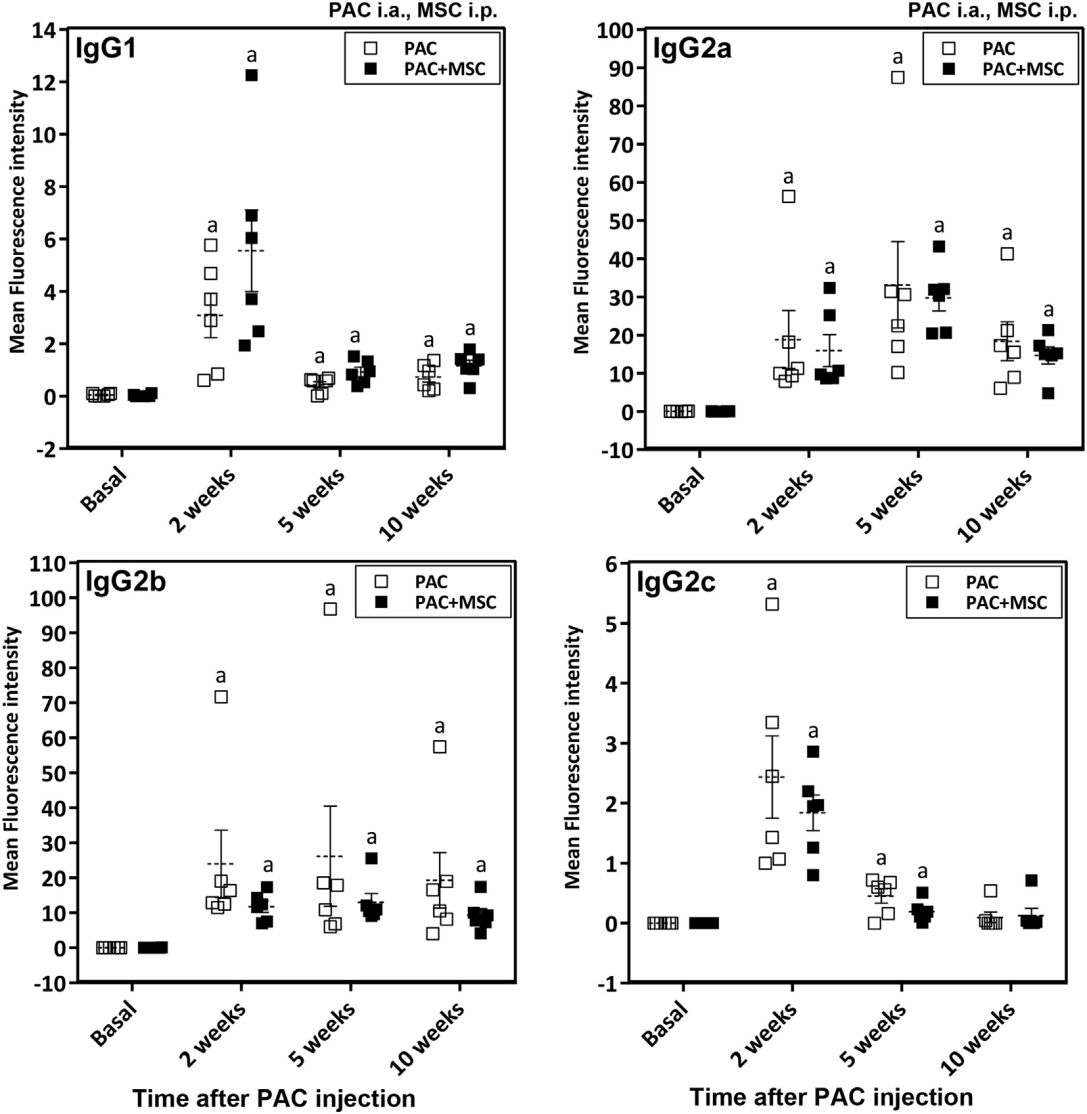
Changes in IgG antibody subtypes in Lewis rats injected intraarticularly (i.a.) with porcine articular chondrocytes (PAC) only or posttreated with mesenchymal stem cells (MSC) intraperitoneally (i.p.). The study included samples from the PAC and PAC + MSC cohorts collected at baseline, 2, 5, and 10 weeks after PAC injection (Figure S1C in Supplementary Material). In particular, the PAC cohort received only PAC i.a., whereas the PAC + MSC rats were injected with MSC i.p. 3 weeks after PAC i.a. injection. Anti-PAC IgG1, IgG2a, IgG2b, and IgG2c antibody reactivity was determined by flow cytometric analysis in sera of all experimental rats at 1%. The mean FL-1 fluorescence intensity after subtracting the background (reactivity of secondary antibody alone) is shown as mean ± SEM for the PAC and PAC + MSC cohorts (*n* = 6). Statistically significant differences were observed using the Wilcoxon test for the assessed time points and subtypes relative to corresponding baseline levels as indicated (^a^*p* ≤ 0.05). No significant differences were observed using the Mann–Whitney *U* test when comparing results from the PAC and PAC + MSC cohorts.

Anti-PAC IgM and IgG responses were detected by flow cytometry in all rats injected with PAC but not in controls (DMEM and MSC cohorts) (Figure 4). Both anti-PAC IgM and IgG showed the highest reactivity at week 2 (first measurement after baseline) and diminished slowly afterward, although remained above baseline levels by week 18. No effect of the MSC (injected at week 3) was observed on the antibody response (Figures 4C,F). The elicited antibodies were further characterized in the cohorts injected with PAC i.a. by measuring the anti-PAC IgG subtypes (Figure 5). The profiles for rats injected with or without MSC were identical. The four IgG subtypes increased after PAC injection, although with different patterns. Changes in anti-PAC IgG1 and IgG2c followed the same timing of transient elevations at week 2 followed by dramatic decreases afterward. On the contrary, anti-PAC IgG2a peaked at 5 weeks and remained high at 10 weeks, whereas anti-PAC IgG2b remained high after the first increase at week 2.

### Injection of PAC i.a. Induced a Xenogeneic Cellular Immune Response Which Was Not Influenced by MSC Administered after 3 Weeks

**Figure 6:**
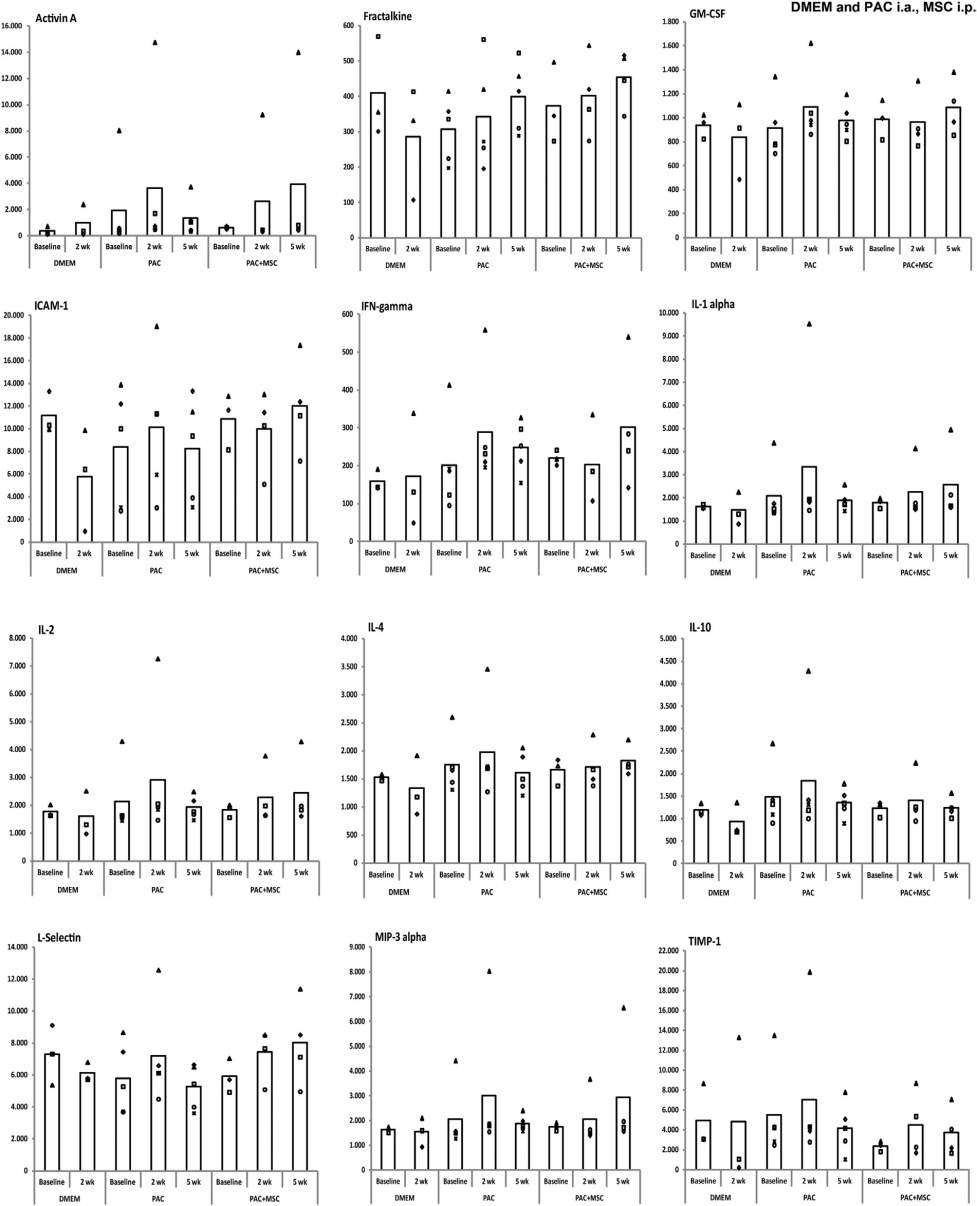
Serum levels of selected markers assessed by antibody array in Lewis rats injected intraarticularly (i.a.) with porcine articular chondrocytes (PAC) only or posttreated with mesenchymal stem cells (MSC) intraperitoneally (i.p.). The study included samples from three of the cohorts described in Figure S1C in Supplementary Material. In particular, one cohort received only DMEM i.a., another only PAC i.a., and the last one was injected with MSC i.p. 3 weeks after PAC i.a. injection (PAC + MSC). Results are presented in reference units. Determinations were done in samples from the DMEM (baseline and 2 weeks, *n* = 3 each), PAC and PAC + MSC cohorts (baseline and at 2 and 5 weeks after PAC injection, *n* = 5 for PAC and *n* = 3/4 for PAC + MSC). Each eight-sample array included samples for all time points from the same individual, with the exception of one baseline sample from the PAC + MSC group, and assessed rats from different cohorts. Thus, each animal within one cohort is depicted with the same symbol and can be followed over time. The bars represent the mean.

Serum levels of 34 proteins were assessed by antibody array (Table 2) in 32 samples from individual rats of the DMEM, PAC, and PAC + MSC cohorts including at baseline and at 2 and 5 weeks. No differences were observed for most proteins tested, but results corresponding to 12 markers associated with inflammation and T-cell responses (activin A, fractalkine, GM-CSF, ICAM-1, IFN-γ, IL-1α, IL-2, IL-4, IL-10, L-selectin, MIP-3α, and TIMP-1) seemed potentially modified by the treatments (Figure 6) and were further evaluated. Thus, data from the PAC and PAC + MSC cohorts at baseline and week 2 were combined because an identical protocol was applied before week 3. Results and statistical analyses of the data pooled (*n* = 8) and shown for 10 of these proteins in Table 3 allowed to appreciate a distinct pattern between the DMEM group and the cohorts receiving PAC. Whereas most of these selected markers remained unchanged or even diminished in the vehicle-injected cohort, there was a profile associated with increases in serum marker levels after PAC injection at week 2 (Figure 6; Table 3). By week 5 postinjection, all these proteins had returned to baseline levels with exception of fractalkine (Figure 6). IL-2 and L-selectin were significantly elevated 2 weeks after PAC injection (*p* = 0.036, *n* = 8). Nonetheless, IL-1α was also significantly elevated, whereas GM-CSF and ICAM-1 showed similar trends toward significance. The effect of MSC on the markers was less clear, but it was still of interest to compare the profiles of the PAC and PAC + MSC cohorts at 2 and 5 weeks to assess the presence of some counteractive trends (Figure 6).

**Table 2:** Proteins identified in RayBio^®^ rat cytokine antibody array G2.

Symbol	Full protein name
Activin A	Inhibin beta A chain, homodimer
Agrin	Agrin
CD86 (B7-2)	CD86 antigen
Beta-NGF	Beta-nerve growth factor
CINC-1	Chemokine (C–X–C motif) ligand 1 (CXCL1)
CINC-2	Chemokine (C–X–C motif) ligand 3 (CXCL3)
CINC-3	Chemokine (C–X–C motif) ligand 2 (CXCL2)
CNTF	Ciliary neurotrophic factor
FasL	Fas ligand (CD178, CD95L, TNFSF6)
Fractalkine	Fractalkine (CX3CL1)
GM-CSF	Granulocyte-macrophage colony-stimulating factor (CSF2)
ICAM-1	Intercellular adhesion molecule 1 (CD54)
IFN-gamma	Interferon gamma
IL-1 alpha	Interleukin-1 alpha (IL-1 F1)
IL-1 beta	Interleukin-1 beta (IL-1 F2)
IL-1 R6	Interleukin-1 receptor-like 2 (IL-1 Rrp2)
IL-2	Interleukin-2
IL-4	Interleukin-4
IL-6	Interleukin-6
IL-10	Interleukin-10
IL-13	Interleukin-13
Leptin	Leptin (LEP)
LIX	Chemokine (C–X–C motif) ligand 5 (CXCL5)
L-Selectin	L-selectin (CD62L)
MCP-1	Chemokine (C–C motif) ligand 2 (CCL2)
MIP-3 alpha	Chemokine (C–C motif) ligand 20 (CCL20)
MMP-8	Matrix metalloproteinase-8
PDGF-BB	Platelet-derived growth factor subunit B (PDGFB)
Prolactin R	Prolactin receptor
RAGE	Advanced glycosylation end product-specific receptor
TCK-1	Chemokine (C–X–C motif) ligand 7 (CXCL7)
TIMP-1	Metalloproteinase inhibitor 1
TNF alpha	Tumor necrosis factor alpha

**Table 3:** Serum levels of selected markers assessed by antibody array in Lewis rats injected i.a. with PAC.

Biomarker	Cohort and time			
	DMEM baseline	DMEM 2 weeks	PAC^a^ baseline	PAC^a^ 2 weeks
GM-CSF	937 ± 36	839 ± 113	943 ± 77	1,049 ± 99^b^
ICAM-1	11,190 ± 649	5,749 ± 1,588	9,320 ± 1,531	10,678 ± 1.679^c^
IFN-γ	159 ± 10	173 ± 53	209 ± 34	259 ± 48
IL-1α	1,616 ± 29	1,474 ± 250	1,966 ± 354	2,989 ± 984^d^
IL-2	1,779 ± 77	1,609 ± 285	2,019 ± 334	2,757 ± 692^d^
IL-4	1,529 ± 19	1,329 ± 189	1,714 ± 143	1,919 ± 243
IL-10	1,192 ± 47	936 ± 129	1,388 ± 194	1,737 ± 387
L-selectin	7,288 ± 661	6,125 ± 211	5,831 ± 630	7,585 ± 859^d^
MIP-3α	1,635 ± 38	1,547 ± 207	1,943 ± 360	2,708 ± 803
TIMP-1	4,957 ± 1,136	4,867 ± 2,586	4,321 ± 1,346	63,74 ± 2,060

*Data from the PAC and PAC + mesenchymal stem cells (MSC) cohorts were combined because an identical protocol was applied before week 3. Results are presented as mean ± SEM of the array reference units*.

*^a^Data correspond to the PAC and PAC + MSC cohorts pooled together (*n* = 8) and compared with baseline levels by Wilcoxon signed-rank test*.

*^b^Statistical analysis versus baseline values, p = 0.069*.

*^c^Statistical analysis versus baseline values, p = 0.09*.

*^d^Statistically significant versus baseline values, p ≤ 0.05 for IL-2, L-selectin, and IL-1α*.

*PAC, porcine articular chondrocytes*.

Coculture assays were set with PAC and PBL isolated from these rats at 2 and 10 weeks postinjection for IL-2 measurements (Table 4). This type of coculture provided information on the level of T-cell activation and co-stimulatory capacity (with ConA) in the presence and absence of PAC. The data showed for most rats and time points that there was some T-cell reactivity after coculture with PAC, which was further enhanced in the presence of ConA. Interestingly, the amount of IL-2 secreted in coculture was lower for PBL from PAC-injected rats than in those not exposed to PAC, although the co-stimulatory signals were preserved (elevations with ConA). Moreover, rats injected with MSC alone showed a profile very similar to the PAC cohort. Nevertheless, no major differences were observed between the PAC and PAC + MSC cohort at this level that could indicate an additional beneficial effect provided by MSC injected 3 weeks after PAC (Table 4, week 10). Very similar results were obtained when we analyzed PBL from three rats collected at week 5 of the experiment (data not shown).

**Table 4:** IL-2 secretion by PBL isolated from experimental rats cultured alone or with PAC for 24 h.

Time and number of rats	Cohorts	IL-2 (pg/ml) (mean ± SEM) and condition			
		PBL	PBL + ConA	PBL + PAC	PBL + PAC + ConA
2 weeks (*n* = 5)	DMEM	77 ± 28	315 ± 29	455 ± 64	846 ± 136
	PAC	103 ± 36	271 ± 115	146 ± 68^a^	584 ± 138
10 weeks (*n* = 5)	DMEM	75 ± 22	405 ± 35	371 ± 68	581 ± 60
	PAC	130 ± 33	271 ± 27^b^	216 ± 89^b^	484 ± 55
	MSC	61 ± 28	372 ± 48	203 ± 58^c^	492 ± 82
	PAC + MSC	52 ± 28	301 ± 46	194 ± 36^c^	509 ± 44

*PBL from the indicated number of rats were isolated and cultured separately in triplicate. Activation of the primary signal was performed with ConA when indicated*.

*Statistically significant differences were analyzed with the Mann–Whitney U test. Significance was attained for the indicated conditions relative to corresponding determination in the DMEM cohort*.

*^a^p ≤ 0.01*.

*^b^p ≤ 0.05*.

*^c^p ≤ 0.02*.

*No significant differences were observed between the corresponding determinations of the PAC + MSC cohort and the PAC or MSC alone cohorts*.

*MSC, mesenchymal stem cells; PAC, porcine articular chondrocytes; PBL, peripheral blood lymphocytes*.

**Figure 7:**
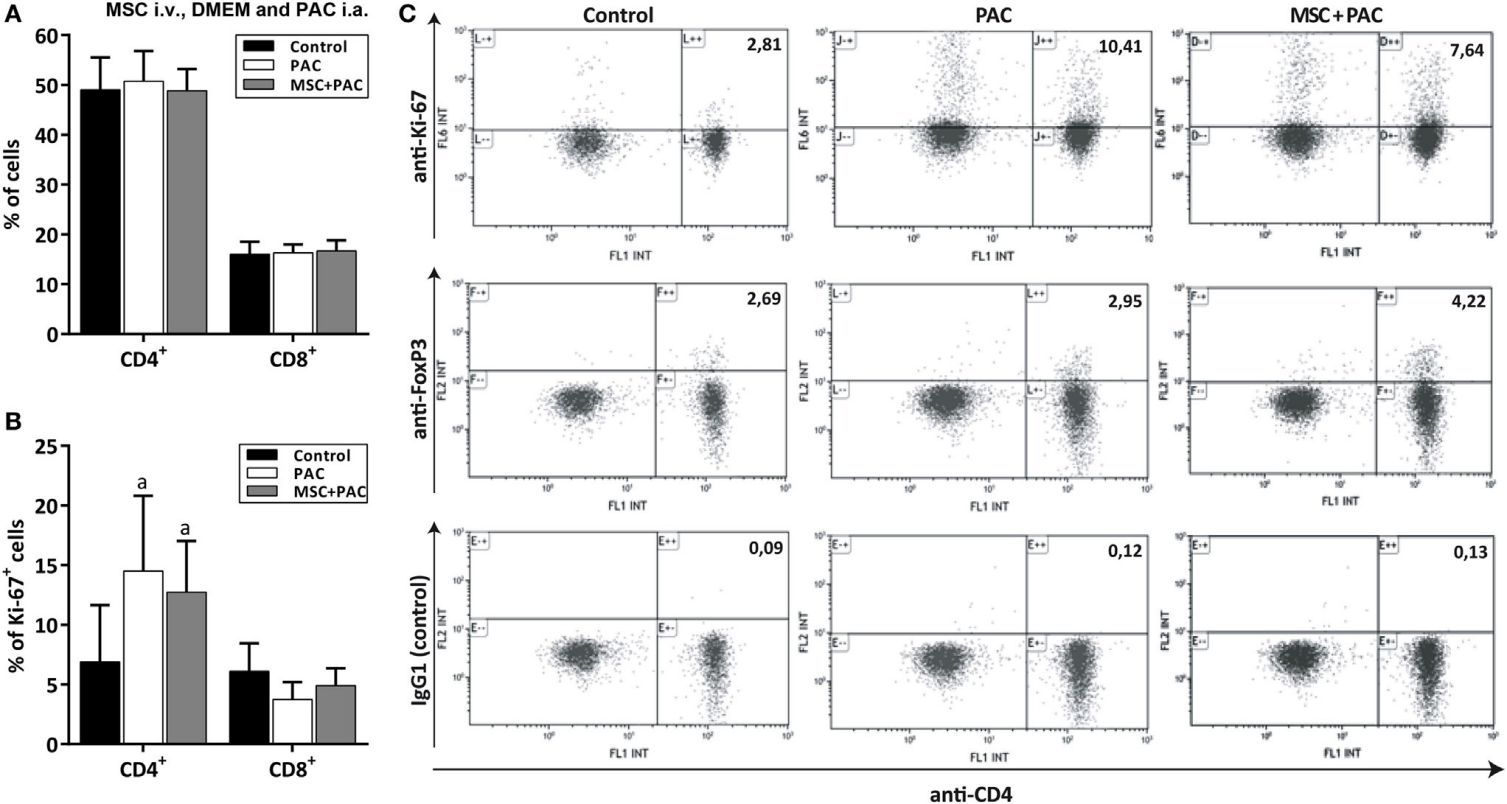
Analysis of CD4^+^ and CD8^+^ lymphocytes isolated from draining lymph nodes from Lewis rats injected intraarticularly (i.a.) with porcine articular chondrocytes (PAC) only or pretreated with mesenchymal stem cells (MSC) intravenously (i.v.). A scheme of the experimental design is shown in Figure S1D in Supplementary Material. In particular, one cohort received only DMEM i.a. as control, another only PAC i.a., and the MSC + PAC was injected with MSC i.v. 1 week before PAC i.a. injection. Flow cytometric two-color analysis of lymphocytes isolated 2 weeks after PAC injection allowed determination of the proportion of CD4^+^ and CD8^+^ cell populations **(A)** and the percentage proliferating by ki-67 expression **(B)** presented as mean ± SEM (*n* = 6). Statistically significant differences were observed using the Mann–Whitney *U* test for the percentage of CD4^+^ki-67^+^ cells relative to the corresponding control cohort (^a^*p* ≤ 0.05), but not for the rest of parameters analyzed. Although only CD4^+^ lymphocytes showed higher proliferation in the PAC-injected cohorts, the proliferation of CD8^+^ T cells may have taken place earlier as it remained in comparable proportion in all cohorts. **(C)** Representative dot plots of anti-CD4 staining in combination with anti-ki-67, anti-Foxp3, and IgG1 control for each cohort (*n* = 6). Numbers indicate the percentage of cells in the upper right quadrant relative to total counts.

In a final study, rats received either DMEM i.a., PAC i.a. or MSC i.v. 1 week before PAC i.a. to assess the cellular immune response at the level of draining lymph nodes 2 weeks after injection (Figure S1D in Supplementary Material). The mean (± SEM) number of total lymphocytes after isolation were, respectively, 2.7 ± 0.37 and 2.87 ± 0.35 million for the PAC and MSC + PAC cohorts *versus* 0.98 ± 0.07 million for the DMEM cohort (*p* = 0.004 for both). Flow cytometric analysis showed that the percentage of CD4^+^ and CD8^+^ lymphocytes remained within the same range for all cohorts (Figure 7A), although CD4^+^ cells showed a higher proliferation as detected by Ki-67 staining 2 weeks after PAC injection (Figures 7B,C). The percentage of Foxp3 cells was not clearly increased after PAC injection (Figure 7C), although most rats pretreated with MSC had higher numbers of these cells (Figure 7C).

## Discussion

In this study, a discordant xenotransplantation model by injecting PAC into the femorotibial joint of Lewis rats was established. A relatively high dose of PAC was chosen considering the amount used for ACI (13, 26) and in previous animal models (5, 27) to generate a setting of high stringency for subsequent testing of immunomodulatory strategies. Notably, this procedure triggered a cellular and antibody response, implying that this simple model may provide valuable preclinical information for the development of xenogeneic therapies for articular cartilage repair. Particularly, it allows assessing strategies intended to modify the immune response such as the procedures studied here based on systemic administration of bone marrow-derived MSC. Conversely, it is not conceived for assessing cartilage reconstruction. Previous models of articular cartilage repair using other species combinations provided mixed results (5, 27, 28). Isolated PAC implanted into a partial chondral defect in rabbits did not induce a cellular immune infiltrate (5). However, rejection was confirmed in a cow-to-rabbit combination when *in vitro* engineered constructs were transplanted into full-thickness lesions subjected to microfracture (28). Furthermore, human articular chondrocytes failed to engraft when transplanted into knee defects in minipigs (27). Of note, none of these studies evaluated antibody response. In comparison, our model provides information at both the cellular and antibody levels in a discordant combination with preclinical value.

Intraarticular injection of PAC elicited an anti-PAC IgM and IgG response, with a peak at week 2 postinjection and predominance of IgG2a and IgG2b subtypes that persisted over time. Notably, rat IgM and IgG2b are potent inducers of complement activation and known to contribute to xenograft rejection of solid organs (29). The transient increase in IgG2c may also participate at this level. We have also determined a role of antibody and complement in PAC activation and triggering an immune response through upregulation of adhesion molecules and cytokine release (10, 11). Regarding their effect on innate cellular immune responses, IgG2a and IgG2b display the strongest effector functions in mice (30). Although we found no information about the affinity of rat IgG subclasses to the various Fc receptors, rat IgG2a and IgG2b show around 80% sequence similarity with the mouse counterparts and 92% for IgG1. Thus, it can be argued that this antibody binding would facilitate the response to PAC by macrophages (that express all activating Fc receptors) and antibody-dependent cell-mediated cytotoxicity by NK cells (expressing FcγRIII). Moreover, the predominance of both IgG2a and IgG2b subtypes indicates the presence of both Th1 and Th2 T cells and associated cytokines. In rats, it is known that Th2 cytokines (IL-4) promote IgG1 and IgG2a responses, whereas Th1 cytokines (IL-12 and IFNγ) stimulate the production of IgG2b and IgG2c (31).

The cytokine/chemokine profiling in serum provided evidence of an active cellular response characterized by elevation of multiple markers at 2 weeks. IL-1α, IL-2, and L-selectin reached significance, whereas other markers showed trends. IL-1 cytokines are produced by monocytes and neutrophils and effect potent pro-inflammatory activity through vasodilatation, recruitment, and activation of immune cells, as well as promotion of matrix degradation (32). Interestingly, serum L-selectin, being generated mostly by shedding, is gaining attention as a marker of cell activation (33). Likewise, ICAM-1 is released to circulation by shedding, whereas TIMP-1 plays the opposite role by controlling the activity of matrix metalloproteinases (34). The increase in GM-CSF is also of interest as it reflects the promotion of myeloid activity including the maturation of pro-inflammatory macrophages (35). Finally, the serum IL-2 augment (characteristic of T-cell activation) paralleled the higher cellularity and proliferation in draining lymph nodes. Non-significant increases in IFNγ, IL-4, and IL-10 were consistent with a balanced Th1/Th2 response in this setting. Most markers shown in Table 2 shared a pro-inflammatory profile.

Cocultures of PAC with PBL isolated from rats without prior exposure to PAC produced some IL-2 secretion that was further enhanced by ConA. The costimulatory molecule CD86 expressed on PAC (8) may be the main trigger for T-cell activation in the presence of ConA. Notably, PBL from PAC-injected rats displayed some degree of T-cell hyporesponsiveness. This finding is consistent with recent experiences reporting the capability of chondrocytes to favor immune regulation and privilege through various pathways (36–38). Thus, production of nitric oxide and induction of indoleamine 2,3-dioxigenase contributed to this effect (36, 38). Moreover, we found no clear increase in regulatory T cells after PAC injection that could lead to hyporesponsiveness. Although xenogeneic chondrocytes are more immunogenic than allogeneic and autologous chondrocytes (28), they probably share some (if not all) the tolerogenic pathways of autologous chondrocytes. Thus, genetic engineering of PAC may allow reducing the immunogenic molecules while preserving the immune privilege capabilities.

Systemic administration of allogeneic MSC under the two selected conditions, both based on single injections, did not prevent or reduce cellular and antibody responses against PAC. Furthermore, T-cell hyporesponsiveness was not enhanced in the cohorts additionally receiving MSC. The time points of MSC injection chosen for this work aimed to avoid the highest peak of inflammation and the potential deleterious effects as previously described for solid organ allotransplantation (20, 39–41). In fact, Merino et al. have recently shown in the same Wistar-to-Lewis rat combination that a full week is needed for MSC infused i.v. to generate an anti-inflammatory environment (41). Accordingly, pretreatment with MSC 7 days before renal allotransplantation protects better from acute graft rejection than when MSC are infused 4 days before transplant (41). The dose of MSC was also selected based on previous data reported in transplant studies in Lewis rats (39, 42). Moreover, the MSC were administered i.p. for the main experiment because the commonly used i.v. route led to short-lived MSC, which died entrapped in the lung capillaries (43, 44). By contrast, the MSC administered i.p. survived much longer and displayed even higher intensity than those injected i.a. Although less frequently used, the i.p. administration of bone marrow-derived MSC was reported to be protective in a model of collagen-induced arthritis (45). We acknowledge that there is no information of superiority of the i.p. route over the i.v. administration. Nevertheless, the relevance of these differences should be taken with caution considering how they can be translated into clinical practice. Importantly, none of the animals injected with MSC outside the joint, either i.v. or i.p., showed MSC migration to the PAC-injected joint, which is similar to results obtained in arthritis mouse models (46).

Some hyporesponsiveness in the MSC control group (injected i.p.) was detected as compared with the vehicle-injected control, whereas the MSC injected i.v. 1 week before PAC increased the presence of Foxp3-positive lymphocytes in draining lymph nodes. Moreover, expression of iNOS was also confirmed in these cells, discarding any defect in our MSC preparation. The efficacy of allogeneic MSC administered i.p. was also related to T-cell hyporesponsiveness, modulation of inflammatory cytokines, and increased activity of regulatory T cells in the collagen-induced arthritis model (45). Similar observations applied to various animal models of autoimmune disease (18), solid organ, and corneal allotransplantation (20, 47) after infusion of MSC. Thus, the stronger immune response triggered by xenografts relative to allografts may explain why MSC failed to potentiate this effect when used in combination with PAC. The fact that we observed a potential enhancement of the immune response with a regime (MSC i.v. day −7) that had previously been described as protective in an allogeneic transplant setting (40, 48) indicated a differential outcome in xenotransplantation. A possible explanation for these results would be that some pro-inflammatory events triggered by the infusion of allogeneic MSC amplify the xenogeneic immune response against PAC. In accordance with higher requirements of immune regulation in the xenogeneic setting, local application of allogeneic MSC failed to prolong survival of corneal xenografts in a pig-to-rat model (48), whereas protection has been described for corneal allografts (47). Nevertheless, this is a concept that has to be demonstrated in well designed experiments that study in parallel the outcome of equivalent allografts and xenografts in well defined settings. Furthermore, the fact that MSC and PAC share some tolerogenic pathways (38) may also contribute. This is also a new hypothesis that is worth considering for future work. Additional immunoregulatory properties of MSC such as promotion of regulatory T cells (16, 20, 47) also remain to be clarified in this setting (49).

Finally, we cannot discard without further experimentation that a higher dose of MSC or a different administration regime (i.e., MSC administration a few days to a week after PAC injection or repeated MSC injection) might exert a stronger immunosuppressive effect. A single injection of allogeneic MSC did not induce a specific antibody response, indicating that an additional dose could be applied. This finding could be associated to this specific allogeneic combination (Wistar-to-Lewis) as other groups have reported the induction of antibodies after allogeneic MSC administration in other animal models including healthy rats (22). Nevertheless, the immunoregulatory capabilities of MSC also include inhibition of plasmablast formation and the associated antibody generation (50). Technical aspects regarding the detection of the elicited alloantibodies may also have influenced. Here, we used for detection of antibody reactivity, as target cells, the same MSC used for injection in the *in vivo* studies. In any case, we do agree that future work should continue to include measurement of anti-donor MSC antibodies as this could be a factor to impact on the efficacy of MSC-based therapies.

## Conclusion

In this work, we demonstrate that PAC-injected i.a. in Lewis rats induces a cellular and humoral immune response. Considering previous studies, this is not an expected result. Interestingly, the allogeneic MSC administered systemically as a single injection in control healthy rats caused some T-cell hyporesponsiveness to PAC and did not induce an anti-MSC antibody response. Nevertheless, the two conditions used here for MSC delivery differentially affected but did not prevent the cellular and humoral immune responses triggered by i.a. injection of PAC. Finally, this rat model allows assessing additional protocols and genetic engineering strategies for the development of xenogeneic cell-based therapies for cartilage repair.

## Ethics Statement

(1) MSC were isolated from bone marrow of 14-day-old male Wistar rats (Animal Service, CHUAC) according to previously published procedures (23). The study protocols were approved by the Animal Ethical Committee of Galicia. (2) Four different animal experiments were carried out that involved intraarticular (i.a.) injection of PAC into the right femorotibial joint of Lewis rats. The protocols were approved by the Ethics Committee of the University of Barcelona and the government of Catalonia.

## Author Contributions

MM, JC, MP-C, PF-P, JF-L, and MA contributed to this work in research design, performance, and analysis; FB, RM, and MA contributed to study conception and design; CC contributed at all levels and wrote the manuscript. All the authors revised and approved the article.

## Conflict of Interest Statement

The authors declare that the research was conducted in the absence of any commercial or financial relationships that could be construed as a potential conflict of interest.
