# Supplementary material for: Biodistribution and Immunogenicity of Allogeneic Mesenchymal Stem Cells in a Rat Model of Intraarticular Chondrocyte Xenotransplantation
Source: Front Immunol. 2017 Nov 6;8:1465. doi: 10.3389/fimmu.2017.01465 (PMC5681521; doi:10.3389/fimmu.2017.01465)
Supplement: Supplementary file 1 [file Presentation_1.PDF]

Supplementary Material

BIODISTRIBUTION AND IMMUNOGENICITY OF ALLOGENEIC MESENCHYMAL STEM CELLS IN A RAT MODEL OF INTRAARTICULAR CHONDROCYTE XENOTRANSPLANTATION.

M. Marquina, J.A. Collado, M. Pérez-Cruz, P. Fernández-Pernas, J. Fafián-Labora, F.J. Blanco, R. Máñez, M.C. Arufe, and C. Costa*.

*** Correspondence:** Corresponding Author: ccosta@idibell.cat

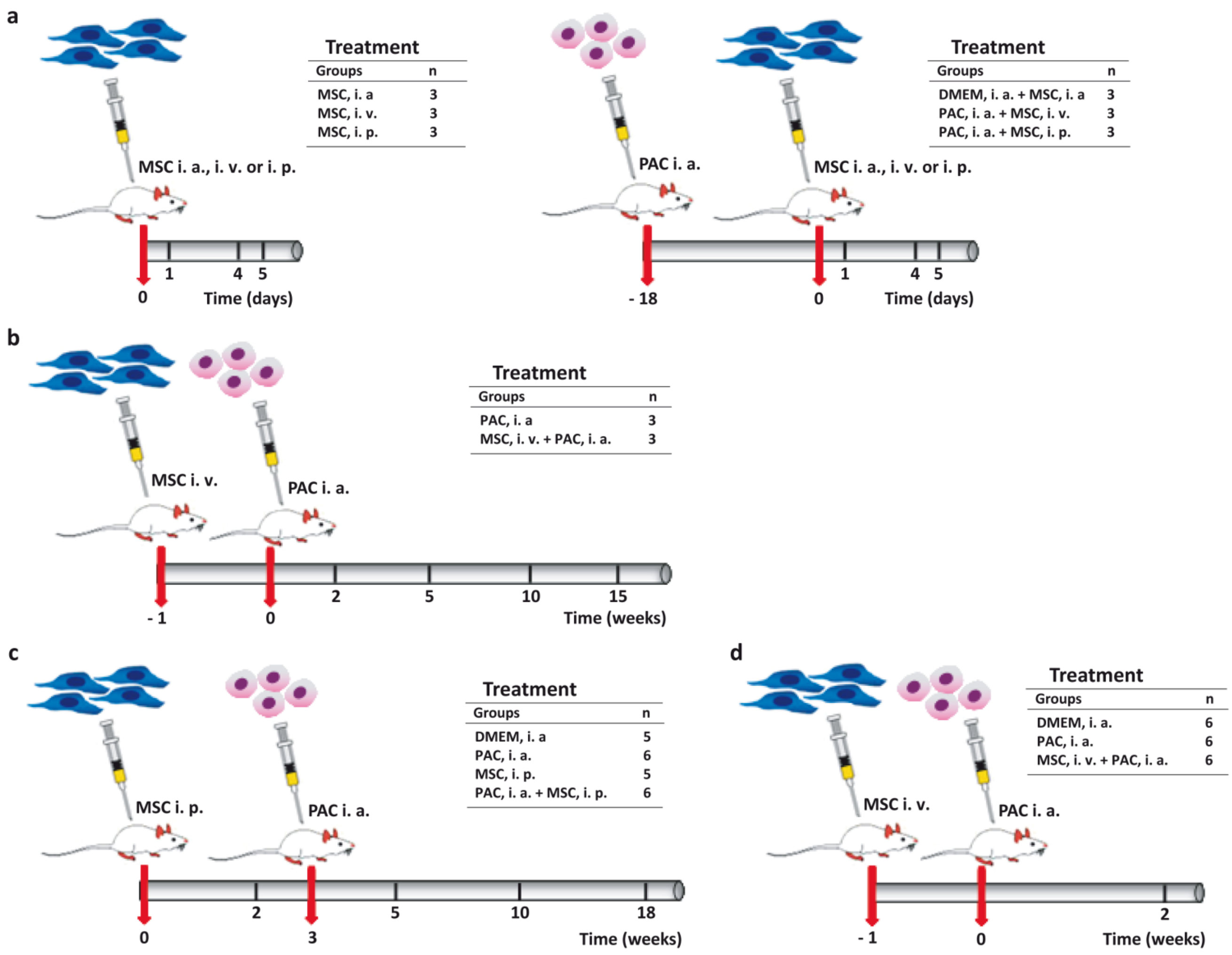

Supplementary Figure 1. Scheme of the experimental animal design. Four different animal experiments were performed which are described in the Materials and Methods section. (**A**) Localization studies of luciferase-labeled MSC in rats untreated or injected with PAC intraarticularly (i.a.) 2.5 weeks earlier. (**B**) Study of the immune response for 15 weeks in rats injected i.a. with PAC only or also pre-treated with MSC intravenously (i.v.). (**C**) Study of the immune response for 18 weeks in rats injected i.a. with PAC only or also post-treated with MSC intraperitoneally (i.p.). Controls of DMEM only or MSC only are included. (**D**) Study of the local immune response in rats injected i.a. with PAC only or also pre-treated with MSC i.v.. Controls of DMEM only are included.

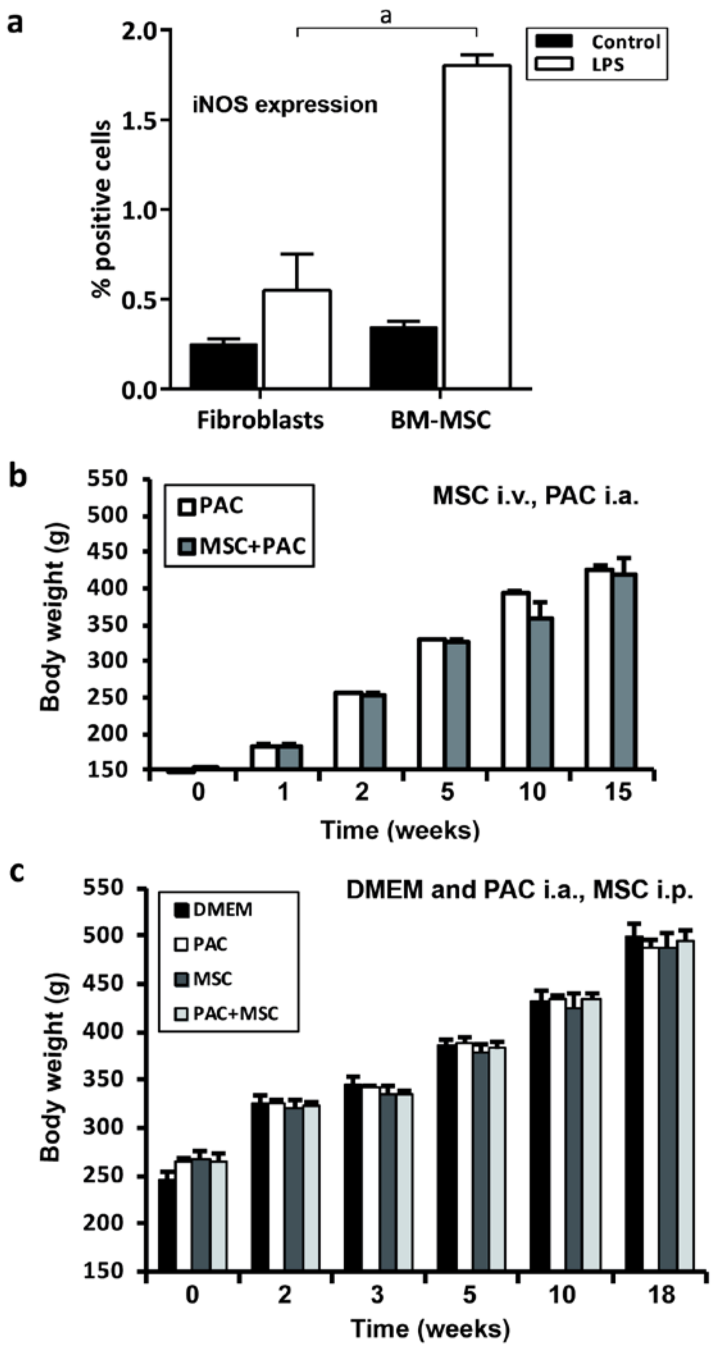

Supplementary Figure 2. Experiment of Lewis rats injected intraarticularly (i.a.) with PAC only or additionally treated with MSC. (**A**) Function of the allogeneic MSC preparations used *in vivo* was assessed *in vitro* by determining the proportion of iNOS-expressing MSC in resting and LPS-stimulated conditions. Murine fibroblasts (3T3L1) were included as control. Data are presented as mean ± SEM (n=4). Statistical differences were observed using the Mann-Whitney U test between LPS-activated MSC and fibroblasts as indicated ($^{a}p \leq 0.02$), but not between resting cells. (**B**) Body weight measurements throughout the 1-week pre-treatment study (suppl. Fig. 1B) for the two experimental groups are presented as mean ± SEM (n=3). (**C**) Body weight measurements throughout the 3-week post-treatment study (suppl. Fig. 1C) for all the experimental groups are presented as mean ± SEM (n=5/6). No statistically significant differences were observed between the experimental cohorts regarding body weight.
